# Demographics, Comorbidities, and Care-Seeking Intent Among Individuals with Obesity or Overweight Status Using Outpatient AI-Based Virtual Triage

**DOI:** 10.1089/tmr.2025.0024

**Published:** 2025-05-22

**Authors:** George A. Gellert, Anna Nowicka, Maria Marecka, Gabriel L. Gellert, Tim Price

**Affiliations:** ^1^ Infermedica, San Antonio, Texas, USA.; ^2^ Infermedica, Wroclaw, Poland.; ^3^ J. Gromkowski Regional Specialty Hospital, Wroclaw, Poland.; ^4^ Clinical Department of Infectious Diseases and Hepatology, Wroclaw Medical University, Wroclaw, Poland.

**Keywords:** obesity, overweight, virtual triage and care referral, artificial intelligence, symptom checker, digital triage, telemedicine, virtual health care

## Abstract

**Objective:**

Compared with persons with normal body mass index (BMI), examine the profile and health care-seeking intent of individuals with obesity/overweight status engaging outpatient artificial intelligence-based virtual triage and care referral (VTCR).

**Methods:**

VTCR encounters of patients with high and normal BMI were compared over a 56-month period to assess differences in demographics, clinical risks, symptoms, conditions, triage recommendations, and care intent.

**Results:**

In 7,222,363 encounters, 29.6% of patients reported having obesity/overweight status, increasing with age and peaking at 45–59 years (46.4%). Mean age for the high BMI group was 35.2 years and 28.7 years in the normal BMI group. Patients with obesity/overweight status reported noncommunicable diseases twice as frequently, including hypertension (relative risk [RR] 2.6), hypercholesterolemia (RR 2.4), diabetes mellitus (RR 2.4), and asthma (RR 1.4) (*p* < 0.05). The group of individuals with obesity/overweight status frequently reported musculoskeletal disorders and gastroesophageal reflux, chronic fatigue symptoms, and were up to four times more likely to have hypertension, obstructive sleep apnea, chronic renal disease, chronic heart failure, cholecystolithiasis, and peripheral vascular disease (*p* < 0.05). Patients with high BMI were slightly more likely to receive triage recommendations for urgent outpatient consultation or emergency department evaluation. Over one-third of patients were uncertain about the appropriate level of care to engage, but this decreased by half (56.6%) following VTCR in both groups.

**Conclusions:**

VTCR effectively identified individuals with high BMI and their associated comorbidities. The results suggest that patients with obesity/overweight status utilize health care services at higher rates. VTCR holds promise as a valuable patient engagement, screening, early diagnosis, and health monitoring tool in managing obesity/overweight status in populations.

## Introduction

Obesity or overweight status and their associated disease risks have become a leading public health challenge. In 2022, 2.5 billion adults (18 years and older) were overweight (body mass index or BMI ≥25 kg/m^2^).^1^ Of these, 890 million were living with obesity (BMI ≥30 kg/m^2^).^1^ This is projected to rise to 3.3 billion people or 37% of the world’s population by 2035.^2^ Prevalence of obesity alone (BMI ≥30 kg/m^2^) will increase from 14% to 24%, affecting nearly 2.1 billion people by 2035. The obesity pandemic confronts nations across all income levels. In high-income countries, the adult obesity rate is projected to reach 37% for women and 42% for men by 2035 (up from 28% and 29%, respectively, in 2020).^2^ Lower-income countries had dramatic increases over the past decade, and obesity prevalence is expected to double by 2035, from 5% to 11% among males and from 14% to 26% among females.^3^

Obesity is widely recognized as a chronic, progressive condition, distinguishing it from acting solely as a risk factor for other diseases.^3^ Obesity substantially increases the risk of noncommunicable diseases (NCDs), including metabolic diseases (diabetes mellitus type 2 and metabolic dysfunction associated steatotic liver disease), cardiovascular diseases (hypertension, myocardial infarction, and cerebrovascular stroke), musculoskeletal (MSK) diseases (osteoarthritis), Alzheimer’s disease, and depression. Obesity also heightens risk of leading malignancies such as breast, colon, prostate, ovarian, liver, and renal cancer.^4^ The health burden of obesity, whether considered as an independent condition or as a contributing risk factor for NCDs, reduces life expectancy by 5 to 20 years.^4^ Obesity also has significant negative economic impact. According to the World Obesity Atlas 2023, obesity reduces global gross domestic product by 2.4%, which is expected to increase to 2.9% by 2035.^2^ Economic costs of obesity nearly equal those due to the 2019 coronavirus pandemic, which contracted the global economy by 3% in 2020.^2^

Obesity management can be supported by current information technologies. Mobile applications supporting behavioral changes to foster weight loss are widely used.^5–10^ Technology-based weight loss tools demonstrated strong adoption and weight loss outcomes comparable to those achieved with minimal in-person interventions, but reported higher rates of weight regain compared with in-person methods.^7^ Digital and e-health tools have assisted patients undergoing bariatric surgery^8^ and aided clinicians and patients as a decision-support tool.^9,10^ An important potential use of digital and e-health tools is for screening symptoms and complications associated with obesity, and managing population health among patients with high BMI.

Artificial intelligence (AI)-based virtual triage and care referral (VTCR) has emerged as a technology to help address challenges in managing patients with obesity or who are overweight. In recent years, public use of online symptom checkers or VTCR has grown in popularity as they offer easily accessible remote health care, providing patients with access to automated evidence-based clinical triage and care guidance. AI-based VTCR has demonstrated an ability to improve the clinical appropriateness of acuity-level care intentions and decisions after patients were triaged that were sustained in post-triage care-seeking behavior, with 35% of patients altering their care plans to match the evidence-based recommendation of virtual triage.^11^ VTCR demonstrated effectiveness in early detection of high incidence life-threatening conditions, indicating a potential to reduce diagnostic and care delays which negatively impact clinical outcomes.^12^ This study is one of the first to profile VTCR patients with obesity/overweight status and to compare their comorbidities, conditions, and care intentions with those of individuals with normal BMI.

## Methods

### Study objective

To evaluate whether demographics, self-reported clinical risks, chief complaints and symptoms, VTCR output conditions, and pre- and post-triage care intent differed systematically between VTCR patients reporting obesity/overweight (high BMI) status compared with normal BMI users.

### Study design

A retrospective cohort study collected and analyzed patient-user reported data from a free online VTCR engine, Symptomate (from Infermedica, Denver, USA), over a 56-month period.

### Setting and description of intervention/virtual triage engine utilized

The Infermedica Symptomate VTCR engine is designed for free public use and deploys AI to conduct evidence-driven evaluations for 800 diseases, 1500 symptoms, and 200 risk factors. VTCR evaluates symptoms shared online by patients. Utilizing machine learning and natural language processing, the VT engine assesses patient-user reported symptoms, seeks more information as needed, evaluates varied clinical hypotheses and possibilities, and indicates the most likely conditions based on the patient-user medical history and clinical presentation. After assessing symptom presentation and medical history, the VTCR AI identifies conditions that most closely align with the patient presentation and input, conveys information about the nature and potential consequences of each condition, and refers the patient to the safest acuity-level appropriate clinical care: self-care, outpatient physician visit (urgent within 24 h or routine), emergency department (ED) care. VTCR can be a standalone free application on the internet, like Symptomate, or it can be integrated with the patient engagement, intake, telemedical, and appointment processes within a particular health system or health plan. There is no way to implement this on technology a multinational or even multistate basis at the present time, unless there is a single care and information system and architecture within a particular geographic or service area. The free online VTCR engines presumes that patients will engage their existing health plan and care services for non-urgent and routine levels of care acuity, and will proceed to the nearest ED if warranted.

AI-based VT engines require rigorous validation to assure patient safety and to minimize potential mistriage. By design, VTCR focuses on common diseases, with the integral AI developed to err on the side of over-triage to higher acuity care, rather than potentially missing and misguiding a patient with acute care needs. VTCR accuracy varies across care specialties and delivery settings, as shaped by the breadth and depth of disease-specific data and content that was used to train the triage AI. VTCR validity has been assessed using a range of clinical vignettes prepared by physicians of varied patient clinical/symptomatic presentations in different clinical settings.^13–17^ Infermedica’s virtual triage engine provides safe recommendations in 97.8% of instances.^15^ Published studies, while providing a point in time comparison, become quickly outdated due to the rapid evolution of AI-based VT.

Virtual triage technologies (including Symptomate) are considered medical device class I in Europe according to Medical Device Directive (93/42/EEC). In the United States, VTCR is regulated under the Food, Drug, and Cosmetic Act. The Food and Drug Administration (FDA) currently exercises enforcement discretion and has determined that VTCR technology is not required presently to comply with FDA regulations related to medical devices.

### Sample selection and eligibility criteria

Data were extracted from the free online Symptomate application with over 19 million Symptomate-completed VTCR encounters and clinical evaluations. The sample consisted of 7,222,363 patient-user VTCR encounters completed during a 56-month period between January 2020 and August 2024. Because VTCR use is anonymous and deidentified, there was no way to differentiate if any particular encounter was with a unique patient, or was a repeat encounter of a prior patient-user. Study participants were selected according to the following eligibility criteria: (1) encounters reporting obesity or overweight status (2,138,755 encounters); (2) encounters where obesity or overweight status was not indicated (5,083,608); (3) encounters where sex and age were recorded; and (4) all patients were 1 year of age and older. Users below age 18 years are encouraged to request a parent or guardian to navigate VTCR on their behalf, and it is assumed that the large majority of encounters with children as denoted by patient age are actually engaged by a parent or guardian seeking guidance about a condition or symptoms impacting a child.

VTCR patient-users provided explicit consent prior to and as an integral step in every virtual triage encounter for their data to be used in a deidentified manner in aggregate analyses for research purposes. All data in this report were analyzed and are presented in a fully deidentified, anonymous manner.

### Data captured and analyses completed

Analyses evaluated if clinical risks, chief complaints, symptoms, conditions, and pre- and post-triage care intent differed systematically between VTCR patients reporting obesity/overweight status compared with normal BMI users. Obesity/overweight status was self-reported through a risk questionnaire during the VTCR encounter. The dataset was compared for the frequency of the top three clinical conditions generated by VTCR per encounter between the two groups. To address significant demographic differences between groups, a sample weighting method was utilized so that weighted samples were not significantly different from each other in terms of age and gender, enabling meaningful comparison of reported clinical comorbidities and conditions, reported clinical symptoms, VTCR condition and care referral output, and pre- and post-VTCR intent. Statistical significance of differences was evaluated using a Z-test with *p* value < 0.05.

## Results

### Patient-User demographics by obesity/overweight status

A total of 7,222,363 VTCR encounters (unique or repeat) were completed during the study period, with 29.6% (2,138,755) of patients reporting obesity/overweight (high BMI) status. Females constituted 67.0% of the total sample, and 30.4% of all females had obesity/overweight status compared with 28.0% of males (Table 1). Similar gender distribution was observed in each BMI group. In the high BMI arm of the study, females comprised 68.8% of the sample and males 31.2%; in the normal BMI arm, females accounted for 66.2% and males 33.8%.

**Table 1.** Distribution of Patients with Obesity, Overweight, and Normal BMI Status by Gender

	Obesity/overweight high BMI status (% gender high BMI)	Normal BMI status (% gender normal BMI)	All patients (% of entire sample)
Female	1,472,135 (30.4%)	3,366,927 (69.6%)	4,839,062 (67.0%)
Male	666,620 (28.0%)	1,716,681 (72.0%)	2,383,301 (33.0%)
Total (% of entire sample)	2,138,755 (29.6%)	5,083,608 (70.4%)	7,222,363 (100.0%)

BMI, body mass index.

The age groups most commonly represented in both the high and normal BMI categories were 18–29 and 30–44 years, accounting for 75.7% of patients with high BMI and 83.2% of patients with normal BMI. However, individuals with obesity/overweight status tended to be older than those in the normal BMI group, with a higher proportion of patients with obesity/overweight status falling into age groups above 30 years compared with their normal BMI counterparts (*p* < 0.05). Fewer than 1 in 10 patients under 18 years old reported having obesity/overweight status, whereas nearly half (46.4%) of those aged 45–59 years did (Table 2). Mean or average age in the high BMI group was 35.2 years compared with 28.7 years in the normal BMI group (*p* < 0.05). By the third decade of life (ages 18–29), a fifth of all patients report high BMI, and then from age 30 onward obesity/overweight prevalence doubled to about two in five over the remaining years of life.

**Table 2.** Distribution of Patients with Obesity, Overweight, and Normal BMI Status by Age

	Obesity/Overweight high BMI status (% of age stratum)	Normal BMI status (% of age stratum)	All patients (% of entire sample)
<18 years old	26,996 (1.3%)	245,856 (4.8%)	272,852 (3.8%)
18–29 years old	847,204 (39.6%)	3,033,644 (59.7%)	3,880,848 (53.7%)
30–44 years old	771,693 (36.1%)	1,192,695 (23.5%)	1,964,388 (27.2%)
45–59 years old	374,877 (17.5%)	432,892 (8.5%)	807,769 (11.2%)
>59 years old	117,985 (5.5%)	178,521 (3.5%)	296,506 (4.1%)
Total of all age groups	2,138,755 (100.0%)	5,083,608 (100.0%)	7,222,363 (100.0%)

BMI, body mass index.

### Patient-user language

To ensure confidentiality and anonymity, Symptomate does not record the national location of patient-user but does capture their selected language, offering a choice of 16 different languages. Although English, Spanish, German, French, and Polish were the five most commonly selected languages in both groups, the distribution of languages differed significantly between the two groups. Specifically, a higher proportion of English speakers (+5.2%) was observed among patients with obesity/overweight status than in the normal BMI group (50.0% vs. 44.8%, *p* < 0.05); in the normal BMI group, there were slightly more French-speaking patients (+1.4%). All other BMI group differences in language use were less than a single percentage point (PP) in magnitude.

### Comorbidities reported by patients

Comorbidities were more commonly reported by patients having obesity/overweight status relative to the normal BMI group, comorbidities more than double for hypertension, hypercholesterolemia, and diabetes (*p* < 0.05). Diagnosed hypertension was reported by 14.7% of the high BMI group versus 5.6% of the normal BMI group, with a relative risk (RR) of 2.6, followed by hypercholesterolemia (10.5% vs. 4.4%; RR 2.4) and diabetes (2.8% vs. 1.2%; RR 2.4; Table 3). Patients with high BMI were 1.4 times more likely to report asthma and were 1.1 times more likely to be peri- and postmenopausal (*p* < 0.05). In the high BMI group, the mean age at which postmenopausal status was reported was 56.1 years, compared with 58.0 years in the normal BMI group (*p* < 0.05).

**Table 3.** Distribution of Patients with Obesity, Overweight, and Normal BMI Status by Clinical Comorbidity/Condition

	Obesity/overweight high BMI status (%) (*N* = 2,138,755)	Normal BMI status (%) (*N* = 5,083,608)	Relative risk (RR) and absolute difference (percentage points/PP)^a^
Hypertension	314,876 (14.7%)	285,322 (5.6%)	2.6 RR (9.1 PP)
Hypercholesterolemia	224,706 (10.5%)	226,074 (4.4%)	2.4 RR (6.1 PP)
Diabetes mellitus (all types)	60,076 (2.8%)	59,030 (1.2%)	2.4 RR (1.6 PP)
Asthma	30,812 (1.4%)	51,621 (1.0%)	1.4 RR (0.4 PP)
Menopausal stage of life	78,327 (3.7%)	171,525 (3.4%)	1.1 RR (0.3 PP)

^a^ All differences were statistically significant at *p* < 0.05.

BMI, body mass index.

### Symptoms reported by patients

Patients with high BMI most frequently reported MSK complaints (14.1%, RR 1.2), gastroesophageal reflux (3.3%, RR 1.4), and chronic fatigue (3.2%, RR 1.2; *p* < 0.05; Table 4). The largest differences relative to patients with normal BMI were in the frequency of reported MSK symptoms (2.1 PP, 1.2 RR) and weight gain (1.0 PP, RR 3.2).

**Table 4.** Distribution of Patients with Obesity, Overweight, and Normal BMI Status by Reported Clinical Symptoms

	Obesity/overweight high BMI status (%) (*N* = 2,138,755)	Normal BMI status (%) (*N* = 5,083,608)	Relative risk (RR) and absolute difference (percentage points/PP)^a^
Musculoskeletal complaints^b^	300,837 (14.1%)	611,908 (12.0%)	1.2 RR (2.1 PP)
Gastroesophageal reflux	69,726 (3.3%)	121,217 (2.4%)	1.4 RR (0.9 PP)
Chronic fatigue^c^	68,030 (3.2%)	137,246 (2.7%)	1.2 RR (0.5 PP)
Abdominal pain	45,352 (2.1%)	81,211 (1.6%)	1.3 RR (0.5 PP)
Weight gain	32,270 (1.5%)	24,230 (0.5%)	3.2 RR (1.0 PP)

^a^ All differences were statistically significant at *p* < 0.05.

^b^ Includes back pain, joint pain, musculoskeletal pain, and paresthesia.

^c^ Fatigue lasting more than 6 months.

BMI, body mass index.

### AI-based triage-automated clinical assessment

VTCR clinical condition output varied significantly between groups. Table 5 displays decreasing RR of 13 common conditions by BMI level. Patients with high BMI were four times more likely to have symptoms consistent with obstructive sleep apnea (1.8% vs. 0.5%; *p* < 0.05). Other conditions with statistically significant and clinically meaningful higher prevalence in the high BMI group included chronic renal disease (1.1% vs. 0.5%; RR 2.3), type 2 diabetes (1.2% vs. 0.6%; RR 2.0), hypertension (0.8% vs. 0.4%; RR 2.0), and chronic heart failure (2.6% vs. 1.4%, RR 1.8).

**Table 5.** Distribution of Patients with Obesity, Overweight, and Normal BMI Status by VTCR Condition Output

	Obesity/overweight high BMI status (%) (*N* = 2,138,755)	Normal BMI status (%) (*N* = 5,083,608)	Relative risk (RR) and absolute difference (percentage points/PP)^a^
Obstructive sleep apnea	38,946 (1.8%)	22,929 (0.5%)	4.0 RR (1.3 PP)
Chronic renal disease	22,966 (1.1%)	23,549 (0.5%)	2.3 RR (0.6 PP)
Diabetes mellitus type 2	25,559 (1.2%)	30,587 (0.6%)	2.0 RR (0.6 PP)
Hypertension	16,702 (0.8%)	19,823 (0.4%)	2.0 RR (0.4 PP)
Chronic heart failure	54,779 (2.6%)	73,273 (1.4%)	1.8 RR (1.2 PP)
Cholecystolithiasis	35,402 (1.7%)	46,515 (0.9%)	1.8 RR (0.8 PP)
Peripheral vascular disease	11,806 (0.6%)	15,652 (0.3%)	1.8 RR (0.3 PP)
Gastroesophageal reflux disease	57,805 (2.7%)	81,572 (1.6%)	1.7 RR (1.1 PP)
Nephrolithiasis	63,368 (3.0%)	107,058 (2.1%)	1.4 RR (0.9 PP)
Asthma	24,886 (1.2%)	41,825 (0.8%)	1.4 RR (0.4 PP)
Joint disorders and overuse syndromes^b^	71,727 (3.4%)	131,498 (2.6%)	1.3 RR (0.8 PP)
Autoimmune diseases^c^	52,603 (2.5%)	103,356 (2.0%)	1.2 RR (0.5 PP)
Acute coronary syndromes^d^	51,939 (2.4%)	106,403 (2.1%)	1.2 RR (0.3 PP)

^a^ All differences were statistically significant at *p* < 0.05.

^b^ Includes osteoarthritis, rotator cuff syndrome, carpal tunnel syndrome, shoulder impingement syndrome, and greater trochanteric pain syndrome.

^c^ Includes rheumatoid arthritis, systemic scleroderma, and psoriatic arthritis.

^d^ Includes myocardial infarction and unstable angina pectoris.

BMI, body mass index; VTCR, virtual triage and care referral.

### AI-based virtual triage-automated care referral

Patients with high BMI were slightly more likely to receive a VTCR recommendation for urgent outpatient consultation within 24 h and evaluation in an ED (Table 6). Differences among patients with high versus normal BMI are minor, with all less than a single PP except for the self-care group.

**Table 6.** Distribution of Patients with Obesity, Overweight, and Normal BMI Status by VTCR-Recommended Clinical Triage Level

VTCR-recommended care acuity level	Obesity/overweight high BMI status (*N* = 2,096,022)	Normal BMI status (*N* = 5,088,349)	Absolute difference percentage points/PP (*p* value)
Self-care	12.0%	13.2%	1.2 PP (*p* < 0.05)
Routine outpatient consultation	30.8%	31.1%	0.3 PP (*p* < 0.05)
Urgent outpatient consultation (in 24 h)	27.7%	27.3%	0.4 PP (*p* < 0.05)
Emergency department	29.5%	28.4%	1.1 PP (*p* < 0.05)

BMI, body mass index; VTCR, virtual triage and care referral.

### Patient-user care-seeking intent prior to and following VTCR encounters

Care-seeking intent was compared by BMI for patients who completed optional pre- and post-VTCR care-seeking intent surveys, including 77,961 in the high BMI and 173,788 in the normal BMI groups. Table 7 compares the reported care intent of each BMI group prior to and following the VTCR encounter, and between groups. Care intent change differences between the two BMI groups are minimal, equal to or less than 1 PP. Largest post-VTCR changes observed were very similar between BMI groups: a mean 14.8 PP (41.2%) increase in intent to engage self-care, and a mean 19.9 PP (56.6%) reduction in patients uncertain of their post-VTCR care intent. Table 7 shows that prior to and following the VTCR encounter, patients with high BMI reported slightly lower intent to engage self-care and slightly higher intent to visit an ED or seek a routine outpatient consultation than those with normal BMI (*p* < 0.05). Over one-third of all patients, regardless of BMI, had uncertain pre-VTCR care intent. Findings were similar in the post-VTCR triage intent survey.

**Table 7.** Pre- to Post-VTCR Care Intent Change Among Patients with Obesity, Overweight, and Normal BMI Status

Care acuity level	Obesity/overweight high BMI status (*N* = 77,961)			Normal BMI status (*N* = 173,788)		
	Pre-VTCR intent	Post-VTCR intent	Care intent change percentage and % points/PP (*p* value)	Pre-VTCR intent	Post-VTCR intent	Care intent change percentage and % points/PP (*p* value)
Self-care	34.8%	49.4%	+14.6 PP [+42.0%] (*p* < 0.05)	36.2%	51.1%	+14.9 PP [+40.9%] (*p* < 0.05)
Routine outpatient consultation	26.3%	28.7%	+2.4 PP [+9.2%] (*p* < 0.05)	24.7%	27.9%	+3.2 PP [+12.6%] (*p* < 0.05)
Urgent outpatient consultation (in 24 h)	1.6%	1.6%	0 PP [+5.3%]	1.4%	1.6%	+0.2 PP [+11.1%] (*p* < 0.05)
Emergency department	2.5%	4.6%	+2.1 PP [+83.0%] (*p* < 0.05)	2.3%	4.4%	+2.1 PP [+88.2%] (*p* < 0.05)
Uncertain care intent	34.8%	15.7%	−19.1 PP [−55.1%] (*p* < 0.05)	35.2%	15.1%	−20.1 PP [−57.2%] (*p* < 0.05)

BMI, body mass index; VTCR, virtual triage and care referral.

## Discussion

Patients with obesity, overweight, and high BMI status comprised over one-fourth (29.6%) of the 7.2 million individuals encounters sampled, less than the figure estimated by the World Health Organization.^1^ This may reflect the study population being younger than many national populations, with 84.7% under age 45 and 57.5% under 30. Mean age in the high BMI group was 35.2 years, whereas the U.S. mean age of individuals with obesity is 46.8 years for men and 48.4 years for women.^18^ The mean age of all VTCR patients (30.4 years) is lower than the general U.S. population (39.2 years).^19,20^ Given that obesity/overweight status increases with age, oversampling of younger VTCR users may have contribute to the lower prevalence of obesity/overweight status observed. However, with the increasing prevalence of childhood obesity, the mean age of individuals with obesity may be declining.^21^ Patients were also disproportionately (two-thirds) female, aligning with prior studies of this VTCR engine.^19^ The fact that English-speaking users reported a 5.2% greater rate of having obesity/overweight status relative to normal BMI may reflect that this language segment predominantly consisted of Americans, where obesity rates are high.

The most frequently reported comorbidities among VTCR patients with obesity/overweight status were hypertension, hypercholesterolemia, diabetes mellitus, and asthma, each associated with and exacerbated by obesity, highlighting the crucial contribution of high BMI to their prevalence.^22–35^ Clinical conditions output by VTCR confirmed the well-documented increased RR of NCDs associated with elevated BMI, including obstructive sleep apnea, diabetes mellitus type 2, hypertension, chronic heart failure and renal disease, cholelithiasis, peripheral vascular disease, gastroesophageal reflux disease, nephrolithiasis, joint disorders/overuse syndromes, autoimmune diseases, and acute coronary syndromes.^22–44^ These findings are interesting given the sample having more females and lower relative age, as several of these conditions in the general population skew toward older males. High BMI women reported being menopausal 2 years younger than normal BMI counterparts (56 vs. 58 years old). Evidence regarding whether obesity contributes to a reduction in reproductive years remains inconclusive. While studies have demonstrated that obesity may be associated with earlier onset of menopause,^45–47^ others do not corroborate this finding^48,49^ or suggest delayed onset of menopause in women with obesity.^50^

With respect to change in patient-user care intent following VTCR, the findings are similar for high and normal BMI groups. Meaningful changes in care intent were noted across all levels of care acuity except urgent outpatient consultation within 24 h. Thus, VTCR is as effective in redirecting individuals with obesity/overweight status to a more appropriate care acuity level as it is for normal BMI patients. Favorable increases in post-VTCR self-care intent and decreased uncertainty about care needed suggest that VTCR empowers patients to confidently take care of themselves, rather than reflexively seeking unneeded higher acuity care (regardless of BMI). VTCR reduced by half (56.6%) the number of individuals who did not know or were uncertain about what kind/level of care to engage. Albeit modest, the post-VTCR increases in routine outpatient and ED care intent in both groups are also important given the ability of VTCR to increase early detection of serious conditions that risk substantial long-term morbidity and mortality.^12^

The observed prevalence of leading NCDs appears low relative to the general population of Western nations. We suspect that this discrepancy reflects lower NCD prevalence among younger females, who are overrepresented in the VTCR user population. For example, the U.S. Centers for Disease Control and Prevention reported that 14.7% of American adults have diabetes; however, only 4.9% are aged 18–44 (closer to the 1.2% reported here).^51,52^ Advanced age is a risk factor for multiple NCDs (hypertension prevalence increases to 71.6% of adults aged 60 and older).^53^ Prevalence of diabetes is higher in men (15.4% vs. 14.1% in women).^51,52^ Rates are also higher in men for hypertension (50.8% vs. 44.6%) and obstructive sleep apnea (33.9% vs. 17.4%), respectively.^53,54^ Peripheral vascular disease affects males 60 years and older at a 10% greater rate than women.^55^ These age and gender differences may explain in part the low NCD prevalences observed in this study. Patients may also be unaware of existing disease and undiagnosed because of younger age and/or other factors, and if asymptomatic, undetected by VTCR.

A study limitation is the lack of clinical verification of conditions identified by AI-based VTCR. Future studies including clinical confirmation of VTCR condition outputs are essential. Also, high BMI does not always indicate obesity/overweight status, for example, in certain athletes. Obesity/overweight status, symptoms, risk factors, and comorbidities evaluated were self-reported and so rely on user accuracy; however, systematic bias is likely mitigated by the large sample size. Lastly, the VTCR knowledge base is grounded in peer-reviewed data on symptoms, risk factors, and conditions—including the impact of obesity/overweight status—which may introduce systematic bias, mitigated by its scale of nearly 100,000 unique connections and consistent application across all concepts.

## Conclusions

VTCR was effective in screening for self-reported obesity/overweight status among patients, with the highest rate reported among those aged 45–59 years. Given that high BMI is associated with substantial avoidable morbidity, mortality, and health care utilization, the ability of VTCR to increase clinically appropriate post-triage self-care, routine outpatient and emergency care is promising. Compared with normal BMI individuals, patients with obesity/overweight status reported significantly elevated rates of comorbidities such as hypercholesterolemia, hypertension, and diabetes mellitus, and more frequently presented with MSK concerns, gastroesophageal reflux, chronic fatigue, and abdominal pain. VTCR output confirmed that patients with obesity/overweight status have higher incidence of conditions such as obstructive sleep apnea, chronic renal disease, type 2 diabetes, hypertension, congestive heart failure, and cholecystolithiasis. Both VTCR outputs and self-declared patient-user intentions suggest that individuals with obesity and overweight status tend to utilize more health care resources compared with their normal BMI counterparts, and more readily seek outpatient consultations and ED care.

These findings suggest that automated AI-based VTCR offers effective population health engagement and utility in health surveillance and monitoring of existing or manifesting chronic and acute disease states, and in detection of obesity-related risk, sequelae, and imminent conditions. With the advent of innovative therapeutics such as glucagon-like peptide 1 to reduce obesity, new vehicles for identifying and engaging individuals with obesity and overweight status can be of substantial potential value in reaching patients in need. Furthermore, VTCR reduced by half the percentage of all patients uncertain about the appropriate level of care acuity to engage, increased post-triage intent to use appropriate outpatient and emergency services, and can contribute to effective management of patients with diverse existing chronic diseases and imminent risks.

## Abbreviations Used

AI: artificial intelligence
BMI: body mass index
EEC: European Economic Community
ED: emergency department
FDA: Food and Drug Administration
MSK: musculoskeletal
N: number
NCDs: noncommunicable disease
PP: percentage points
RR: relative risk
VTCR: virtual triage and care referral
